# A cluster randomized controlled trial comparing Virtual Learning Collaborative and Technical Assistance strategies to implement an early palliative care program for patients with advanced cancer and their caregivers: a study protocol

**DOI:** 10.1186/s13012-021-01086-3

**Published:** 2021-03-11

**Authors:** Lisa Zubkoff, Kathleen Doyle Lyons, J. Nicholas Dionne-Odom, Gregory Hagley, Maria Pisu, Andres Azuero, Marie Flannery, Richard Taylor, Elizabeth Carpenter-Song, Supriya Mohile, Marie Anne Bakitas

**Affiliations:** 1 Division of Preventive Medicine, Department of Medicine, University of Alabama at Birmingham, Birmingham, AL USA; 2 Birmingham/Atlanta VA Geriatric Research Education and Clinical Center (GRECC), Department of Veterans Affairs, Birmingham, AL USA; 3 Dartmouth Hitchcock Medical Center, Lebanon, NH USA; 4 Department of Psychiatry, Geisel School of Medicine, Hanover, NH USA; 5 School of Nursing, University of Alabama at Birmingham, Birmingham, AL USA; 6 Division of Gerontology, Geriatrics and Palliative Care, UAB Center for Palliative and Supportive Care, Department of Medicine, University of Alabama at Birmingham, Birmingham, AL USA; 7 O’Neal Comprehensive Cancer Center, Birmingham, AL USA; 8 University of Rochester Medical Center, Rochester, NY USA; 9 Department of Anthropology, Dartmouth College, Hanover, NH USA

**Keywords:** Implementation strategies, Implementation science, Early palliative care, Advanced cancer, Cluster-randomized controlled trial

## Abstract

**Background:**

Virtual Learning Collaboratives (VLC), learning communities focused on a common purpose, are used frequently in healthcare settings to implement best practices. Yet, there is limited research testing the effectiveness of this approach compared to other implementation strategies. This study evaluates the effectiveness of a VLC compared to Technical Assistance (TA) among community oncology practices implementing ENABLE (Educate, Nurture, Advise, Before Life Ends), an evidence-based, early palliative care telehealth, psycho-educational intervention for patients with newly diagnosed advanced cancer and their caregivers.

**Methods:**

Using Reach, Effectiveness, Adoption, Implementation, Maintenance (RE-AIM) and Proctor’s Implementation Outcomes Frameworks, this two-arm hybrid type-III cluster-randomized controlled trial (RCT) will compare two implementation strategies, VLC versus TA, among the 48 National Cancer Institute Community Oncology Research Program (NCORP) practice clusters that have not historically provided palliative care to all patients with advanced cancer. Three cohorts of practice clusters will be randomized to the study arms. Each practice cluster will recruit 15–27 patients and a family caregiver to participate in ENABLE. The primary study outcome is ENABLE uptake (patient level), i.e., the proportion of eligible patients who complete the ENABLE program (receive a palliative care assessment and complete the six ENABLE sessions over 12 weeks). The secondary outcome is overall program implementation (practice cluster level), as measured by the General Organizational Index at baseline, 6, and 12 months. Exploratory aims assess patient and caregiver mood and quality of life outcomes at baseline, 12, and 24 weeks. Practice cluster randomization will seek to keep the proportion of rural practices, practice sizes, and minority patients seen within each practice balanced across the two study arms.

**Discussion:**

This study will advance the field of implementation science by evaluating VLC effectiveness, a commonly used but understudied, implementation strategy. The study will advance the field of palliative care by building the capacity and infrastructure to implement an early palliative care program in community oncology practices.

**Trial registration:**

Clinicaltrials.gov. NCT04062552; Pre-results. Registered: August 20, 2019. https://clinicaltrials.gov/ct2/show/NCT04062552?term=NCT04062552&draw=2&rank=1

Contributions to the literature

- As recommended by oncology professional organizations, this study will promote the use of an evidence-based, telehealth, early palliative care program for patients with advanced cancer among a national sample of community oncology practices.
- Improve understanding of effectiveness of two commonly used implementation strategies, the Virtual Learning Collaborative (VLC) and Technical Assistance (TA).
- Evaluates measures of ENABLE implementation, organizational factors that influence fidelity to implementation, and patient and caregiver outcomes.
- Lessons learned will inform measurement of multi-level implementation outcomes related to early palliative care interventions.
- Lessons learned will inform future use of telehealth interventions and virtual implementation strategies.

## Introduction

### Background and rationale

Due to the benefits of early palliative care (EPC) in randomized controlled trials [1–3], the American Society of Clinical Oncology recommends “ … combined standard oncology care and palliative care … early in the course of illness for any patient with metastatic cancer and/or high symptom burden” [4] (p.881). Yet, the National Cancer Institute (NCI)’s “Landscape Study” revealed that only 30% of community oncology practices have access to palliative care multidisciplinary teams in outpatient settings [5, 6]. Even practices offering EPC are often unable to meet the needs of all advanced cancer patients due to a limited palliative care workforce and the large number of patients with advanced cancer and caregivers [7, 8]. Training existing oncology staff to provide evidence-based palliative care interventions [9] is a potentially scalable and sustainable approach to address the national shortage of palliative care specialists. The ENABLE (Educate, Nurture, Advise, Before Life Ends) program is an example of such an intervention that can be implemented by healthcare organizations seeking to develop or enhance palliative care services.

**Fig. 1:**
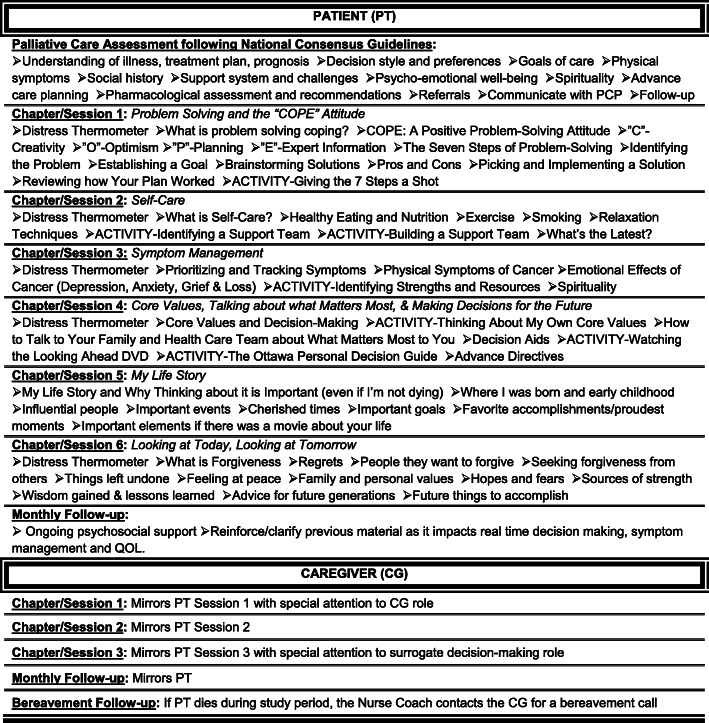
Charting your course (CYC) chapter/session topics

ENABLE is an evidence-based, scalable model of EPC promoted by the National Cancer Institute Evidence-Based Cancer Control Programs (EBCCP), formerly known as Research-Tested Intervention Program (RTIP) [10–12]. ENABLE is led by a Nurse Coach and includes a comprehensive palliative care assessment, weekly telehealth coaching sessions (6 for patient; 3 for caregiver) using the Charting Your Course© guidebook. The coaching sessions review essential palliative care topics, such as problem solving, coping, symptom management, self-care, communication, decision-making, and advance care planning and monthly follow-up calls (Fig. 1). The parallel ENABLE program topics for caregivers mirror the patient sessions. ENABLE has not been widely used in community oncology practices despite being publicly available through the EBCCP. An effective implementation strategy to address implementation barriers (e.g., lack of knowledge about evidence-based EPC, insufficient infrastructure) is needed to equip oncology practices to develop the skills to implement and sustain ENABLE. However, it is unclear which strategy would be most successful in assisting implementation.

Two common strategies to enhance implementation of evidence-based practices include VLC and TA. VLC is an innovative version of the traditional (face-to-face) Learning Collaborative, developed by the Institute for Healthcare Improvement (IHI) to guide clinical teams in implementing evidence-based practices [13]. Learning collaboratives use group interaction among teams to guide clinical improvement efforts. VLC, which uses a virtual (web-based) platform instead of face-to-face meetings, was designed to decrease the cost of participation and increase the speed of incorporating lessons into practice [14, 15]. The evidence on VLCs appears to improve processes, but is inconclusive on outcomes [16, 17].

The second strategy, TA, is defined as “interactive support that is individualized to the specific needs of individuals or teams.” (p.3) [18]. TA is typically provided one-on-one to a single organization and does not use a community structure to encourage shared learning or collaboration [19]. The evidence on TA effectiveness is minimal [19, 20]. To our knowledge, there are few rigorous trials comparing the effectiveness of VLC or TA and more testing with rigorous study design is needed [14, 15, 21, 22]. Which strategy would be most effective for the widespread implementation of ENABLE in community oncology practices is unclear.

To address these gaps in implementation science and palliative care, we developed a cluster randomized controlled trial (RCT) that compares VLC to TA for implementing ENABLE in community oncology practices. Our primary hypothesis is that practices randomized to VLC will outperform those randomized to TA resulting in a higher proportion of patients completing the ENABLE program. The more effective implementation of ENABLE will be due to the benefits of group dynamics (e.g., shared experiences, accountability, learning, discussion, and interactions), and quality improvement learning/skills that occur in the VLC but not in TA. Ultimately, a greater uptake of ENABLE will lead to better outcomes (mood and quality of life) for the patients and caregivers at the practices exposed to VLC compared to those exposed to TA.

### Study aims and hypotheses

**Fig. 2:**
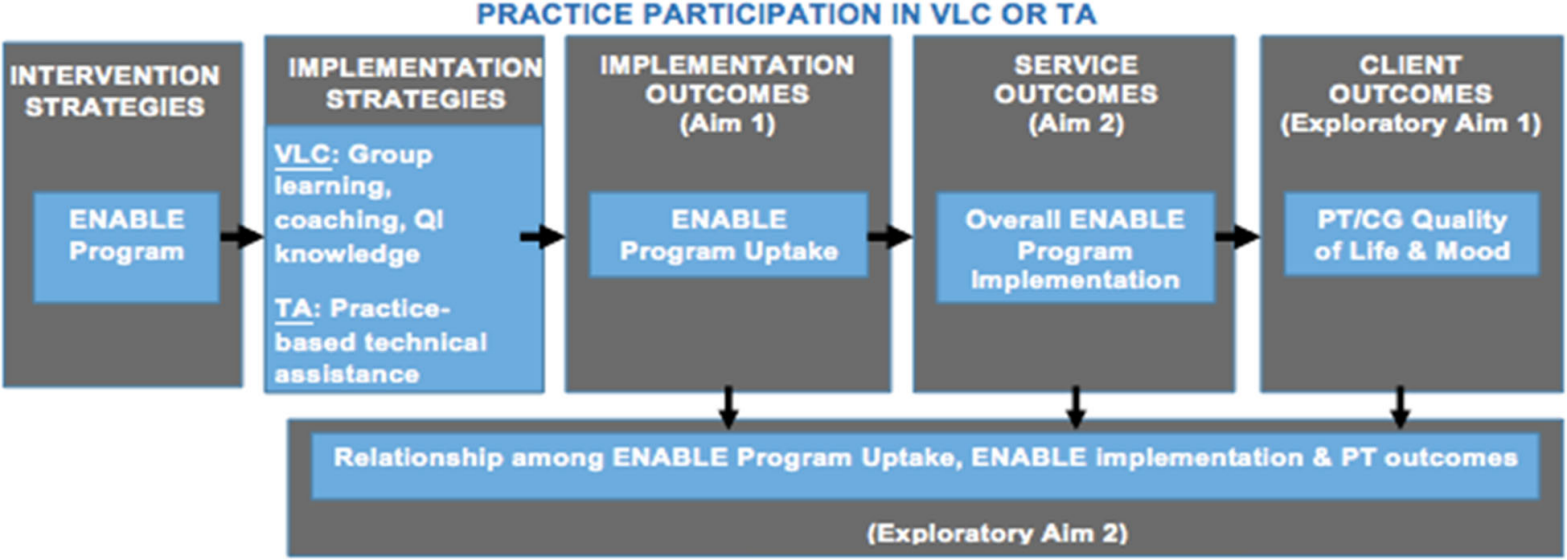
Adapted proctor’s implementation outcomes model applied to study aims

Based on Proctor’s implementation framework, the purpose of this two-arm hybrid type-III cluster RCT in community oncology practices is to compare the effectiveness of two strategies, VLC and TA for implementing the evidence-based ENABLE program (Fig. 2).

### Primary aim (implementation outcome, patient level)

Compare the effectiveness of VLC vs. TA on ENABLE program uptake, as measured by the proportion of patients who complete the ENABLE program (i.e., have a palliative care assessment and complete the six ENABLE sessions). *Hypothesis*: Practice clusters randomized to VLC will have greater ENABLE program uptake than practice clusters randomized to TA.

### Secondary aim (service outcome, practice level)

Compare the effectiveness of VLC vs. TA on practice clusters’ overall ENABLE program implementation. This will be assessed using the ENABLE General Organizational Index (GOI), which measures factors that influence institutional implementation of evidence-based practices. *Hypothesis*: Practice clusters randomized to VLC will have better overall ENABLE program implementation than practice clusters randomized to TA.

### Exploratory aim 1 (patient & caregiver outcomes)

Compare practice clusters randomized to VLC or TA on patient and caregiver quality of life (QOL) and mood outcomes. *Hypothesis*: Patients and caregivers at practice clusters randomized to VLC will have better QOL and mood compared to those at practice clusters randomized to TA.

### Exploratory aim 2

Determine the relationship among ENABLE program uptake, overall ENABLE program implementation, and patients’ QOL and mood across the two strategies. *Hypothesis*: Practice clusters with better ENABLE program uptake and overall ENABLE implementation (regardless of implementation group) will demonstrate a higher degree of improved patient outcomes (i.e., QOL and mood) compared to practice clusters that have low/poor ENABLE program uptake and overall ENABLE implementation.

## Methods/design

### Theoretical framework

This study is guided by the Reach, Effectiveness, Adoption, Implementation, Maintenance (RE-AIM) framework, which is designed to enhance the quality, speed, and public health impact of efforts to translate research into “real world” settings [23, 24]. Further, this study applies Proctor’s Implementation Outcomes framework [25] to assess implementation success via outcomes on three levels: implementation (effects of actions to implement new treatment), service (reflect the practice process changes), and client (evidence-based program’s effect on the target population).

### Trial design

**Fig. 3:**
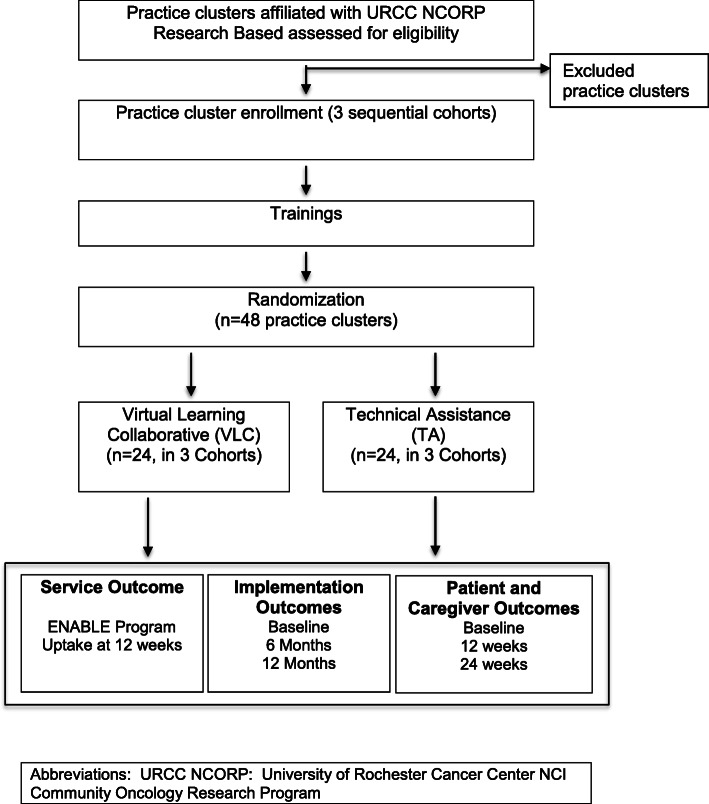
Trial design

This two-arm hybrid type-III cluster RCT compares implementation strategy effectiveness, while gathering additional information on the ENABLE program outcomes [26–28]. VLC and TA have distinct communication channels, time requirements, social systems, and structures [29, 30]. A target sample of 48 NCI’s Community Oncology Research Program (NCORP) practices will be randomized to one of two conditions: 1) VLC group which will receive ENABLE training and participate in a 15-month VLC (*n* = 24) or 2) TA group which will receive ENABLE training and participate in a 15-month TA process (*n* = 24). Each NCORP practice in this study will be formed into “practice clusters.” Practice cluster is defined as an NCORP practice location where oncology physicians and study staff work independently and do not crossover to another practice. Practice clusters will be recruited in 3 cohorts of approximately 16 practice clusters per cohort. In each cohort half of the practice clusters will be randomized to VLC and half will be randomized to TA (Fig. 3).

### Setting/sample

#### URCC NCORP practice cluster participation

This study is being carried out from the University of Rochester Cancer Center (URCC) NCORP Research Base. NCORP is a national network that brings cancer treatment and care delivery trials to people in community settings. NCORP is comprised of 7 Research Bases, and 46 Community Affiliates, each with affiliated practices [31]. The URCC NCORP Research Base is associated with 22 NCORPs and 358 sub-affiliates across the country. URCC NCORP practices vary in size and geographic location; however, to be eligible to participate in this study, they must meet the following inclusion criteria:

- Identify two (or more) Nurse Coaches to deliver the ENABLE program. For practices that only have 1 nurse available, one Nurse Coach is allowed.
- Commitment of Nurse Coaches to be trained to conduct the palliative care assessment and deliver the ENABLE sessions
- Desire to implement ENABLE, including presence of an investigator (e.g., Primary Affiliate Principal Investigator, oncology physician, Cancer Care Delivery Research (CCDR) Lead) and/or program administrator/supervisor who are willing to be key contacts
- Demonstrated support/buy-in from oncology physicians who are willing to enroll patients
- Agreement of practice leadership and staff to support/participate in study activities
- If necessary, willingness to participate in a phone interview to determine capacity to implement the ENABLE program.

##### Exclusion criteria

Practice clusters that currently offer fully functioning inter-professional early palliative care to all cancer patients will be excluded. If practices offer early palliative care services to some, but not to all cancer patients, practices will only enroll patients to this study who have not received early palliative care services.

### Within practice cluster patients and caregivers participants

#### Patients

Patients will be encouraged (but not required) to select a caregiver, defined below, to participate in the study. Participants can receive cancer treatment while participating in the study but will not have received palliative care services at the time of enrollment.

##### Patient inclusion criteria

Patients must be diagnosed with advanced cancer (stage III/IV, recurrence, or progressive solid tumor cancer) within the last 90 days and have an expected survival of at least 6 months. Patients must be English-speaking, at least 18 years old, willing to complete the palliative care assessment and ENABLE sessions, have access to a telephone that can receive calls, and able to provide informed consent to participate in the study.

##### Patient exclusion criteria

Patients who received previous palliative care services will be excluded. However, after enrollment, patients may receive palliative care services outside of the ENABLE program, if appropriate.

#### Caregivers

Each patient can identify a single caregiver to participate in the study. The caregiver is any person who provides support to the patient. Caregivers may remain in the study even if the patient withdraws.

##### Caregiver inclusion criteria

Caregivers are defined as an “unpaid relative or friend who knows them well and who provides regular support to their cancer.” Caregivers must be English-speaking, at least 18 years old, willing to complete the ENABLE Caregiver program sessions, have access to a telephone that can receive calls, and able to provide informed consent to participate in the study.

##### Exclusion criteria

None.

### Practice cluster recruitment

The URCC NCORP Research Base will announce the study via email to all URCC NCORP community affiliates and post study activation on the Cancer Trials Support Unit (CTSU) website. In addition, we will hold information webinars. Interested practice clusters will complete a form to describe the practice (e.g., current palliative care services, demographics of patient population, staff members to be involved in the study) and submit it to URCC Research Base. Each practice cluster will be expected to enroll and deliver ENABLE to 15–27 patients.

### Patient and caregiver recruitment

Staff in each practice cluster will identify eligible patients from clinical schedules and inform oncology physicians. If the physician determines the patient to be eligible and the patient agrees to be informed about the study, study personnel at the practice (e.g., Nurse Coach, research coordinator) will meet the patient either in person or via HIPAA compliant phone or videoconference to review the study. Informed consent will be obtained either in person or using a HIPAA compliant phone or videoconference and documented.

### Evidence-based practice to be implemented: ENABLE

The ENABLE program for patients consists of [1] a standardized in-person or telehealth Palliative Care Assessment [32] and [2] a series of six, 20–60 min weekly telephone coaching sessions provided by a Nurse Coach using the *Charting Your Course©* ***(CYC)*** guidebook [33, 34]. ENABLE fosters patient empowerment [35, 36] through coaching on problem-solving coping, symptom management, self-care, communication, decision-making, and life review and reflection. A Nurse Coach, who is a registered nurse or advanced practice provider (e.g., nurse practitioner or physician assistant) who has at least 2 years’ experience working in oncology or palliative care, delivers the ENABLE program. Caregivers receive a separate series of similar telephone sessions using a CYC*©* guidebook with caregiver-relevant content. After completing the weekly sessions, both the patient and caregiver receive monthly check-in phone calls. Figure 1 presents the CYC*©* session topics. Trials testing ENABLE have demonstrated improved patient quality of life, mood, and survival outcomes and reduced caregiver burden and improved mood [10, 11, 37].

### Implementation interventions (practice cluster level)

Table 1 summarizes the essential elements of the VLC and TA implementation strategies and outlines the distinctive features between the two. Each practice cluster will assemble an implementation team who will participate in the assigned implementation strategy (VLC or TA). At minimum, this includes one or two Nurse Coaches who will deliver the intervention and at least one coordinator who will facilitate recruitment and patient and caregiver outcome assessment. Other members of the practice cluster (e.g., oncologists, data managers) are also encouraged to participate in implementation team and VLC or TA activities.

**Table 1:** Summary of Implementation Strategies

Activity	Virtual Learning Collaborative (VLC)	Technical Assistance (TA)
Form an ENABLE Implementation Team	**✓**	**✓**
ENABLE training	**✓**	**✓**
ENABLE Implementation (15 months)	Group web-based VLC learning sessions (monthly)	Individual practice cluster consultation call with ENABLE expert (monthly)
Coaching/Expert Consultation	**✓**	**✓**
Quality Improvement Knowledge and Information	**✓**	
Individualized Written Feedback	**✓**	
Monthly Group Discussion and Sharing	**✓**	
Between Session Activities	**✓**	

#### Virtual Learning Collaborative

Practice clusters randomized to the 15-month VLC will participate in monthly virtual group-based learning sessions to discuss key components of the ENABLE program, implementation progress (e.g., nurse coach training, number referrals, number patients/caregivers enrolled, sessions completed), data collection activities, and current successes and challenges. In addition, the VLC will include instruction on quality improvement tools and techniques to assist with the implementation process, coaching, and the opportunity to share stories and experiences and learn from other practice clusters using an “all teach, all learn” approach [13]. The VLC will be co-led by ENABLE and quality improvement experts (RT and KDL).

#### TA

Practice clusters randomized to the 15-month TA study arm will receive individual, interactive monthly consultation calls with an ENABLE expert (JND-O). The TA leader will ask similar questions about the practice cluster’s implementation progress as are covered in the VLC study arm (e.g., nurse training, referral process, enrollment, sessions completed, data collection activities, and current successes and challenges). The relationship between the TA provider (JND-O) and practice clusters will be friendly, trusting, strengths-based, collaborative, and will support practice cluster autonomy. We anticipate that practice clusters will solicit input or guidance from the TA leader on challenges or areas of concern. The TA leader will offer advice and suggestions to the degree desired by the practice cluster.

### Study trainings

Nurse Coaches who deliver the ENABLE program will complete the approximately 19-h self-paced 10-module ENABLE training on a web-based platform (Canvas). The training, developed by ENABLE experts, consists of [1] an overview of the ENABLE program; [2] instruction on how to conduct the palliative care assessment and deliver each ENABLE session; [3] role play of the assessment and phone sessions that will be reviewed by an ENABLE expert who will provide feedback; and [4] tools and resources for Nurse Coaches. Upon completion of the ENABLE training, Nurse Coaches will complete an evaluation of the training to receive Continuing Education Unit (CEU) credits.

Additional trainings will cover study procedures and how to use the web-based study platforms: Canvas, Zoom, and Research Electronic Data Capture (REDCap), an electronic data capture tool to enter study data.

### Measures and outcomes

Based on Proctor’s Framework (Table 2), this study has 3 categories of outcomes [1] implementation (aim 1), [2] service (aim 2), and [3] client (patient and caregiver) outcomes (exploratory aim 1).

**Table 2:** Measures and outcomes

Outcome	Instrument(s)	Time Point	Target	Description
***Aim 1 Outcome (Implementation Outcome, Patient Level)***				
ENABLE Uptake	ENABLE Nurse Coach Contact Log (patients and caregivers)	Months 1-15	Coordinator or Nurse Coach	This contact log documents the delivery of each ENABLE session.
***Aim 2 Outcome (Service Outcome, Practice Level)***				
Overall Program Implementation	ENABLE GOI	Baseline, 6, 12 months	Coordinator and Nurse Coach (other staff may attend call)	The ENABLE General Organizational Index (GOI) is conducted via a phone interview with the Coordinator and Nurse Coach (other staff may attend call) to assess factors that influence institutional implementation and fidelity to an evidence-based practice. It covers 12 domains and is scored on a Likert scale. Qualitative analysis will also be conducted [38–40].
*Additional Implementation Outcomes*				
ENABLE Program Implementation	ENABLE RE-AIM Self-Assessment Tool	Baseline, 6, 12 months	Coordinator and Nurse Coach	The ENABLE RE-AIM Self Assessment Tool (Reach, Efficiency, Adoption, Implementation, and Maintenance) was developed in our prior work to collect reach, adoption, implementation, and maintenance [23, 24, 38]. *Reach* is defined as the number of enrolled participants divided by the number of eligible participants. *Efficacy* will be measured by patient and caregiver quality of life and mood outcomes (see Explanatory Aim 1 measures). *Adoption* will be measured by calculating the number of oncology physicians who refer patients divided by those who have the opportunity to refer patients to the study. *Implementation* is captured in the study’s primary outcome, ENABLE uptake. *Maintenance* will be measured by the presence of the ENABLE program 6 months after the completion of the VLC or TA implementation strategy in the ENABLE Sustainment Survey. The ENABLE Nurse Coach and/or coordinator will complete the online ENABLE RE-AIM Self-Assessment tool at baseline, 6 months (± 8 weeks), and 1 year (± 8 weeks).
Perceptions of Early Palliative Care	Provider Perceptions of Early Palliative Care Survey	Baseline, 15 months	Oncologist and Nurse Coach	The survey asks about attitudes about palliative care, feelings about palliative care specialists, comfort with an preferences for providing early concurrent oncology palliative care, and description of early concurrent palliative care provided in the practice cluster [38, 41]. Responses are on a Likert scale.
*VLC and TA Process Outcomes*				
Program Participation and Engagement (VLC or TA)	VLC Practice Participation and Engagement; TA Practice Participation and Engagement	Months 1-15	Study team (VLC lead, TA lead)	The VLC and TA Participation and Engagement forms assess the extent to which the sites participate in the assigned strategy (VLC or TA). The forms share commonalities, but are distinct to reflect the different methods of communicating with practice clusters. The site’s level of participation in the session is scored on a Likert scale with an additional free text entry. A higher score indicates better participation and engagement.
Fidelity (VLC or TA)	VLC Fidelity; TA Fidelity	Months 1-15	Study team (VLC lead, TA lead)	The instrument measures fidelity and adherence to key features of the VLC and TA study arms respectively. The forms will be completed after each session. The instrument for the VLC and TA study arm are distinct. The study team will audit 10% of sessions by reviewing the recording and verifying key components being discussed during sessions.
*ENABLE Process Measures*				
Palliative Care Assessment	Palliative Care Assessment Checklist	Baseline	Nurse Coach	This form documents each of the key components of the palliative care assessment that are completed.
Additional ENABLE Contacts	ENABLE Additional Contacts Log (patients and caregivers)	Months 1-15	Coordinator and Nurse Coach	This instrument tracks other patient and caregiver contacts or activities as part of the ENABLE program. The ENABLE Nurse Coach will document non-session contacts and activities each week (i.e., time spent doing ENABLE related study tasks other than delivering the sessions) and enter them into REDCap. The coordinator will also document the time to complete recruitment and enrollment procedures in the log on REDCap every 2 weeks.
Fidelity to Palliative Care Assessment	Palliative Care Assessment Adjudication Checklist	Baseline, months 4-6	Nurse Coach	An ENABLE expert will review four of each practice cluster’s palliative care assessments to ensure completion and correct documentation. At each practice cluster, we will request the clinical note in the medical record associated with the palliative care assessment for the first two patients enrolled and then a random sample of two additional patients. Study personnel will review the note and compare it to the Palliative Care Assessment Checklist. Adequate fidelity will be demonstrated by ≥85% of the components identified on the Palliative Care Assessment Checklist.
ENABLE Sustainment	ENABLE Sustainment Survey	Month 21	Oncologist and Nurse Coach (may be completed by other staff)	This 9-item instrument measures the presence/sustainment of the ENABLE program 6 months (± 4 weeks) after completion of the 15-month implementation strategy (VLC or TA). Example questions include the number of trained ENABLE Nurse Coaches, number of patient and caregiver participants, ENABLE components currently offered, and any adaptations that have been made to the ENABLE program. Other staff members (e.g., coordinator, Primary Affiliate PI, CCDR Lead) may also complete the survey.
***Exploratory Aim 1 Outcome (Patient Outcomes)***				
Patient Mood	HADS	Baseline, 12, 24 weeks	Patient	The Hospital Anxiety and Depression Scale (HADS) instrument assesses mood, including anxiety and depression. Seven questions rate the depression subscale and 7 questions rate the anxiety subscale. Each item has a 4-point scale, ranging from 0 to 3 with possible scores ranging from 0-21 for each subscale. Scoring for each sub-scale is as follows: 0-7 Normal, 8-10 Borderline abnormal, and 11-21 Abnormal [42–46].
Patient Quality of Life	FACIT-PAL	Baseline, 12, 24 weeks	Patient	The FACIT-Pal consists of the FACT-G (Functional Assessment of Cancer Therapy-General), a general measure of quality of life, and the palliative care subscale (Pal), which assesses issues specifically relevant to palliative care [37, 47–49]. The FACT-G is a 27-item questionnaire that provides a total score as well as four subscale scores: physical, social/family, emotional, and functional wellbeing. The FACIT-Pal includes 19 additional concerns relevant for persons at the end of life. The total score is the sum of the FACT-G plus the FACIT-Pal subscale [50].
*Additional Patient Outcomes*				
Patient Global Health	PROMIS Global Health	Baseline, 12, 24 weeks	Patient	The 10-item measure uses Likert-scale response options for each item, ranging from 1 (always) to 5 (never). This instrument produces 2 scores: physical health and mental health score [51–54].
Symptom burden	MDASI	Baseline, 12, 24 weeks	Patient	The MD Anderson Symptom Inventory (MDASI) is a 19-item measure that assesses symptom severity experienced by patients with cancer and the symptom-related interference with daily living [55, 56]. The MDASI uses a 0-10 severity scale for 13 items, with 0 being symptom “not present” and 10 being “the worst you can imagine,” and an interference 0-10 response scale for 6 items ranging from 0 being “did not interfere” to 10 being “interfered completely”.
Demographic and Clinical Characteristics	Demographics Form	Baseline	Patient	Patients will be asked to report their education level, marital status, whom they live with, employment status, age, gender, ethnicity, race, employment status, diagnosis, insurance status, annual household income, urban vs. rural living location, religious preference, presence of a living companion, prior and current smoking status and usage, prior and current alcohol consumption.
***Exploratory Aim 1 Outcome (Caregiver Outcomes)***				
Caregiver Mood	HADS	Baseline, 12, 24 weeks	Caregiver	See description under Patient Outcomes.
Caregiver Quality of Life	PROMIS Global Health	Baseline, 12, 24 weeks	Caregiver	See description under Patient Outcomes.
Caregiver burden	MBCB	Baseline, 12, 24 weeks	Caregiver	The Montgomery Borgatta Caregiver Burden (MBCB) Scale is a 14-item instrument assesses caregiver burden and the impacts on the caregiver’s life [57–59]. Each item uses a Likert-type scale response option, ranging from 1 (“A lot less”) to 5 (“A lot more”).
Positive Aspects of Caregiving	Positive Aspects of Caregiver	Baseline, 12, 24 weeks	Caregiver	The Positive Aspects of Caregiver is a 9-item instrument that assesses positive aspects of caregiving, including the extent to which the caregiver feels: useful, good about him/herself, needed, appreciated, important, strong and confident, appreciates life, more positive attitude toward life, and strengthened relationships with others. Each item is rated on a 5-point ordinal scale ranging from 1 (disagree a lot) to 5 (agree a lot) [60].
Preparedness for Caregiving	Preparedness for Caregiving Scale	Baseline, 12, 24 weeks	Caregiver	The Preparedness for Caregiving Scale is a self-rated instrument that consists of 8-items that asks caregivers about their perceived preparedness for multiple domains of caregiving, such as providing physical care, providing emotional support, setting up in-home support services, and dealing with the stress of caregiving [61, 62]. Responses are rated on a 5-point scale with scores ranging from 0 (not at all prepared) to 4 (very well prepared) [63]. There is also a question with a free text response.
Demographics	Demographic Form	Baseline	Caregiver	Caregivers will also be asked to report education level, marital status, whom they live with, employment status, age, gender, ethnicity, race, employment status, insurance status, annual household income, urban vs. rural living location, religious preference, relationship to patient participant, if the patient participants lives with them, average number of days per week they help take care of the patient, and average hours per day helping the patient participant.

### Aim 1: ENABLE uptake (implementation outcome, patient level)

The primary outcome will be *ENABLE program uptake* (program completion). To capture ENABLE program uptake, each ENABLE Nurse Coach will use a standardized ENABLE Nurse Coach Contact Log to document patient and caregiver referrals, enrollment, and completion of the essential ENABLE elements (i.e., palliative care assessment, ENABLE sessions). The Nurse Coach Contact Log, which is completed in REDCap, will document each contact and ENABLE session using an assigned study identification for each patient or caregiver.

### Aim 2: Overall ENABLE Program implementation (service outcome, practice cluster level)

This outcome will be measured for each practice cluster via phone interviews with Nurse Coaches and coordinators using the ENABLE General Organizational Index (GOI). This instrument was developed to assess factors that influence institutional implementation of the ENABLE program [38]. We previously adapted [38] the General Organizational Index (GOI) which measures fidelity to an evidence-based practice by assessing two key elements of implementation: individualization and quality improvement [64]. The GOI has been shown to have acceptable psychometric properties [39]. The ENABLE GOI structured interview guide covers 12 domains (program philosophy, eligibility/client identification, penetration, assessment, individualized initial palliative care treatment plan, individualized subsequent ENABLE contacts, supervision, process monitoring, outcome monitoring, quality assurance, and client choices regarding service provision) associated with successful implementation. ENABLE GOI assessments will occur at baseline, 6 months (± 8 weeks), and 1 year (± 8 weeks). After the phone interview, the interviewer [40] will score each domain on a Likert scale for a total range of 12–60. Moreover, interviews will be recorded and transcribed for qualitative analysis to augment quantitative assessment of this outcome.

Table 2 presents additional implementation measures, including RE-AIM, process outcomes for the study arms (VLC and TA), and ENABLE process measures.

### Exploratory aim: patient measures

Table 2 presents patient measures. Patient assessments will be completed at baseline, 12, and 24 weeks. There will be several options to collect the data: [1] study coordinator reads the questions to the patient (either in person or on the phone) and enters the patient responses, [2] the patient completes the assessments in person with a study coordinator, or [3] the patient will take a paper copy home, complete it, and return it at either the next visit, via mail, or scan and email.

### Exploratory aim: Caregiver measures

Caregiver assessments (Table 2) will be completed at baseline, 12 and 24 weeks using the same methods as for patients described above.

### Sample size

#### Aim 1: Implementation outcome (patient level)

The RCT is powered for the aim 1 outcome, ENABLE program uptake, defined as the proportion of patients who complete the ENABLE program. In determining the targeted sample size, we made the following assumptions: [1] an intra-class correlation coefficient (ICC) value of 0.05, [2] alpha of 1% (*α* = 0.01) and 80% power, [3] 20% enrolled patients for whom completion of ENABLE cannot be assessed due to death or becoming too ill to continue with participation, and [4] a proportion of patients completing ENABLE of 0.52 in prior studies. With 24 practice clusters per group and a minimum of 12 patients per practice cluster (down from 15 after 20% unable to assess completion), for a total of 288 patients per group, the detectable difference in ENABLE program uptake is 0.18 (OR = 2.06; RR = 1.33), a medium effect size.

### Randomization

This cluster-randomized trial will be carried out in 3 sequential cohorts of 8–20 practice clusters (4–10 per study arm) to reach the desired sample size of 48 practice clusters. Within each cohort, RCT arm assignment will occur after completion of all required study trainings, including the ENABLE training (Fig. 3). A minimization algorithm for group assignment with random initial start will be used to attempt to simultaneously balance the proportion of rural practices, practice sizes, and minority patients seen within each practice across the two RCT arms. The minimization algorithm will be generated using R software. Study team members and practice cluster members will not be blinded to group assignment or study hypotheses.

### Statistical analysis

All randomized practice clusters will be included in primary comparisons, according to their group assignment, and regardless of their degree of participation in the study. Primary data analysis will include descriptive statistics for baseline practice cluster characteristics, patient characteristics, and outcomes in each RCT arm. We will examine the balance between RCT arms with respect to baseline characteristics (e.g., age, sex, race, cancer type, stage) using effect sizes, such Cohen’s d and Cramer’s. Factors showing non-trivial imbalances between comparators will be used as adjusting covariates in the between-group comparisons. Analysis will be conducted using the latest versions of SAS and R.

#### Analysis for aim 1: Implementation outcome (patient level)

We will use a logit GEE model with exchangeable correlation structure (to account for clustering within practice) for the binary patient uptake indictor (“yes” if the patient completed a palliative care assessment and all ENABLE program sessions prior to the 12 week assessments and “no” if the patient is still alive but has not completed a palliative care assessment and all six ENABLE program sessions prior to the 12-week assessments), with the group assignment as the main predictor. Model-predicted outcome proportions, odds ratios, relative risks, and confidence intervals for these measures will be used for interpretation. Supplemental analyses using appropriate methods for clustered data will consist of between-group comparisons of the proportion of recruited patients who could not complete the ENABLE sessions due to illness or death, and the number of sessions completed among all patients recruited.

#### Analysis for aim 2: Service outcome (practice cluster level)

Analysis will compare VLC vs. TA on NCORP practices’ overall ENABLE program implementation measured by the ENABLE GOI. Due to the small sample size for this practice cluster-level outcome (24 VLC vs. 24 TA), rather than conducting formal inference testing, comparisons will be descriptive statistics and effect size measures.

##### Qualitative analysis

The audio-recorded interviews of the ENABLE GOI will be coded by study team members (EC-S) [40] in Atlas.ti using a qualitative content analysis approach using *a* priori domains from the GOI measure and additional categories that emerge from the inductive review of transcripts [65]. Coded data will be aggregated and reviewed within and across codes to construct an in-depth understanding of facilitators, barriers, and contextual factors that may influence implementation of the ENABLE program.

Findings from the qualitative data will be integrated with the main findings of the RCT at the interpretation and reporting stage [66]. We will use a convergent mixed methods analysis to collect both qualitative and quantitative ENABLE GOI data at roughly the same time, assess information using parallel constructs for both types of data, separately analyze both types of data, and then compare results through discussion and jointly displaying both forms of data. The joint display will map each ENABLE GOI domain score to corresponding concepts or themes from these qualitative findings to more fully understand the context of the GOI domain scores and identify patterns across practice clusters [67]. This will provide validation for each data type and create a foundation for drawing conclusions about the implementation of the ENABLE program [68].

#### Analysis for exploratory aims (patient and caregiver outcomes)

##### Exploratory aim 1

To compare NCORP practices randomized to VLC or TA on patient and caregiver quality of life and mood, linear mixed models will be used to estimate differences in patient and caregiver outcome trajectories over the 24-week follow-up period. If necessary, a false discovery rate adjustment will be used to adjust for multiple exploratory inferences [69].

##### Exploratory aim 2

The association between ENABLE sessions and change in patient outcomes will be examined with linear mixed models. Adjusting covariates will include patient characteristics that may influence participation in sessions (e.g., sex, illness acuity, age). Linear mixed models will also be used to examine the association between GOI scores and patient outcomes.

## Discussion

The relative strengths of learning collaboratives compared to other implementation strategies are poorly understood [16, 17]. This study will provide a direct comparison between the VLC and TA strategies to determine if VLC is a superior method to facilitate implementation of the evidence-based ENABLE program in community-based oncology practices. Finding successful strategies to implement models of early palliative care in cancer is imperative given national guidelines that have highlighted this priority [70].

Most published studies on VLCs are descriptive and have not definitively tested its effectiveness to enhance adoption and implementation [16, 71–82]. There are also few randomized-clinical trials that evaluate traditional in-person learning collaboratives [14, 15, 21, 22] or compare their effectiveness to other implementation approaches [16, 17, 71, 83]. For example, learning collaboratives have been shown to outperform financial incentives or toolkits, but only on some process or secondary outcomes [16, 17]. This study will contribute to the fields of implementation science and palliative care as we will learn about the implementation effectiveness of VLC and TA while delivering an evidence-based practice. While we hypothesize the VLC will outperform TA due to the shared wisdom generated by the group, the VLC strategy has greater demands for sites and facilitators (and possibly costs) and has less opportinuity for individual attention than TA. As such, it is important to assess the added value of each strategy in order to know how to better spread and sustain EPC. Therefore, this study will inform efforts to more widely disseminate ENABLE, while simultaneously advancing implementation science methods and approaches that can be used for other evidence-based practices.

Like most research, we have encountered challenges during the study start-up period. First, the COVID-19 pandemic significantly impacted the ability for NCORP practices to conduct research and resulted in a need to rely on telehealth. Although most ENABLE activities were already offered via telephone, we modified the protocol so that all study procedures can now be conducted remotely, including the consent process and the comprehensive palliative care assessment. During this time, NCORP practices also expressed concern about the time required to complete the Nurse Coach training. To reduce training burden, the length of the web-based ENABLE Nurse Coach training was reduced from 25 h to approximately 19 h. We anticipate the reduced number of training hours may be beneficial beyond the study given our goal to make the ENABLE training more broadly scalable to nurses and advanced practice providers interested in implementing this EPC program. We also amended the originally required cohort size (*n* = 16) and will permit cohorts of 8–20 practice clusters (4–10 per study arm). To account for this in analyses, we will assess for confounding by cohort. Lastly, although each practice cluster will be expected to deliver ENABLE to 15–27 patients, we recognize that may not happen. To account for this, we will include an option to recruit additional practice clusters (up to 20% of 48 which is 58 practices) and additional cohorts. Despite these challenges, we anticipate the first cohort will launch in the first half of 2021.

There are several anticipated lessons learned from this study. First, to the best of our knowledge, this is the first large-scale study measuring the implementation of early palliative care in community-based oncology practices. To measure implementation, we developed and refined palliative care implementation measures and anticipate that lessons learned will be used to improve these measures. Second, this study not only seeks to identify the superior implementation strategy (VLC or TA), but also to examine the key components of each strategy. To do so, we have developed measures of participation, engagement, and fidelity that have not been applied in other published literature. Lessons learned will inform refinement of the measures and advance the field.

### Strengths and limitations

There are several strengths to this study. First, this study will evaluate both the uptake of an evidence-based EPC intervention and organizational factors associated with improving delivery of EPC in community based care. Second is the collaboration with the URCC NCORP. Conducting this work outside a consortium would be difficult, particularly with respect to recruiting practice clusters. Third, this study provides a solution to the limited palliative care workforce by implementing this program as a generalist palliative care model [84]. As part of study activities, Nurse Coaches will be trained on how to deliver the ENABLE EPC program, thereby increasing practice capacity to offer palliative care services [7, 8, 85]. A final, yet unanticipated strength to this study is the opportunity to implement the effective ENABLE EPC telehealth model in community oncology clinics during COVID-19 when telehealth services are particularly important.

While this study will contribute to implementation science methods and approaches, there are limitations. Patients and caregivers will not know which study arm their practice cluster is assigned. However, practice clusters will not be blinded to study aims and hypotheses as sharing those are essential to study recruitment and consent procedures. Similarly, blinding the TA and VLC facilitators and the assessors (e.g., ENABLE GOI interviewer) to the group assignment is not feasible. However, it is feasible to limit their knowledge of specific activities in each study arm to minimize bias. Contamination between practice cluster cohorts is another potential limitation. Since all practice clusters will be affiliated with URCC NCORP, enrolled practice clusters may discuss their study involvement with practices that register for a future cohort. To minimize potential contamination between cohorts, we will instruct second and third cohorts to reach out to the TA or VLC members (depending on randomization assignment) as opposed to outside practices for advice.

## Conclusion

The results from this study will be twofold: [1] we will learn about the implementation of an early palliative care intervention among community oncology practices, and [2] we will learn about the effectiveness of the VLC as compared to TA to facilitate such implementation. In addition, lessons learned will advance the field of implementation science through further refinement of palliative care implementation measures and of the VLC implementation strategy.

## Data Availability

The investigators will provide access to data according to NIH and University of Rochester NCI Community Oncology Research Program policies.

## Abbreviations

ACS: American Cancer Society
CCDR: Cancer Care Delivery Research
CCM: Chronic Care Model
CIC: Chronic Illness Care
CIRB: Central Institutional Review Board
CONSORT: Consolidated standards of reporting trials
CYC: Charting Your Course
DSMC: Data Safety Monitoring Committee
EBP: Evidence-based practice
ENABLE: Educate, Nurture, Advise, Before Life Ends
EPC: Early Palliative Care
ERIC: Expert Recommendations for Implementing Change
FACT-G: Functional Assessment of Cancer Therapy–General
FACIT-PAL: Functional Assessment of Chronic Illness-Palliative Care
GOI: General Organizational Index
HADS: Hospital Anxiety and Depression Scale
ICC: Intra-class Correlation Coefficient
LC: Learning Collaborative
BMCB: Montgomery Borgatta Caregiver Burden Scale
MDASI: MD Anderson Symptom Inventory
NCI: National Cancer Institute
NCORP: NCI Community Oncology Research program
PI: Principal investigator
PROMIS: Patient Reported Outcome Measurement Information System
QOL: Quality of life
RCT: Randomized controlled trial
RE-AIM: Reach, Effectiveness, Adoption, Implementation, Maintenance Framework
REDCap: Research Electronic Data Capture
RUCA: Rural-Urban Commuting Area
TA: Technical Assistance
UAB: University of Alabama at Birmingham
UR: University of Rochester
URCC: University of Rochester Cancer Center
VLC: Virtual Learning Collaborative
